# P-690. Mapping Respiratory Infection Burden in the U.S.: State-Level and Gender-Specific Insights from GBD 2021

**DOI:** 10.1093/ofid/ofaf695.903

**Published:** 2026-01-11

**Authors:** Sameer Kumar Majety, V I nesh Seelam, Komuroju Pooja Mrinmai, Niyaz Shaik, Hemanth Kamadi, Srinivasa Chakradhar Earni

**Affiliations:** School of Medicine, Xiamen University., Kakinada, Andhra Pradesh, India; School of Medicine, Xiamen University., Kakinada, Andhra Pradesh, India; Dr Patnam Mahender Reddy Institute of Medical Sciences, Hyderabad, Telangana, India; School of Medicine, Xiamen University., Kakinada, Andhra Pradesh, India; School of Medicine, Xiamen University., Kakinada, Andhra Pradesh, India; International Higher School of Medicine, Krygyzstan., Hyderabad, Telangana, India

## Abstract

**Background:**

Respiratory infections continue to pose a significant health challenge in the U.S., with disease burdens differing across regions and demographic groups. Using the GBD 2021 dataset, we examined geographic and gender-based disparities in respiratory infection incidence.

**Figure 1:**
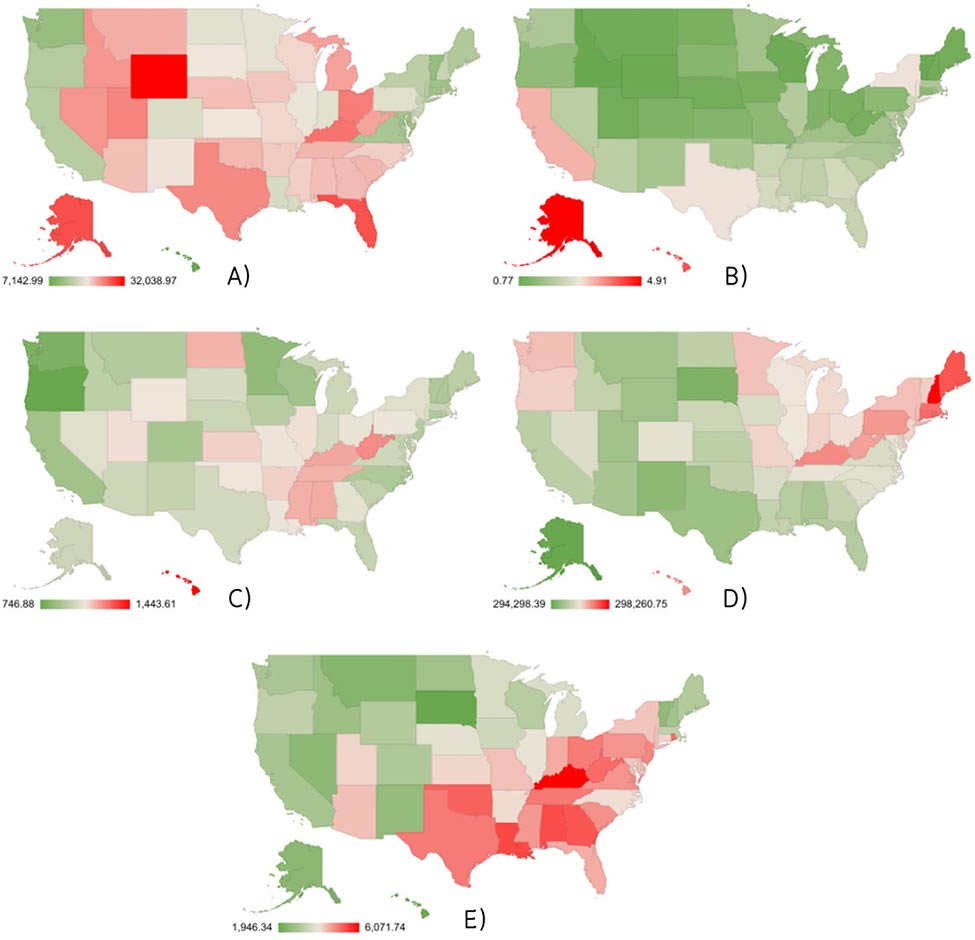
State-wise distribution of respiratory infection and tuberculosis burden in the United States (2021). Panels depict age-standardized incidence rates for (A) COVID-19, (B) tuberculosis, (C) lower respiratory infections, (D) upper respiratory infections, and (E) otitis media. Shades represent relative burden, with red indicating higher values. Data sourced from the Global Burden of Disease (GBD) 2021 study

**Figure 2:**
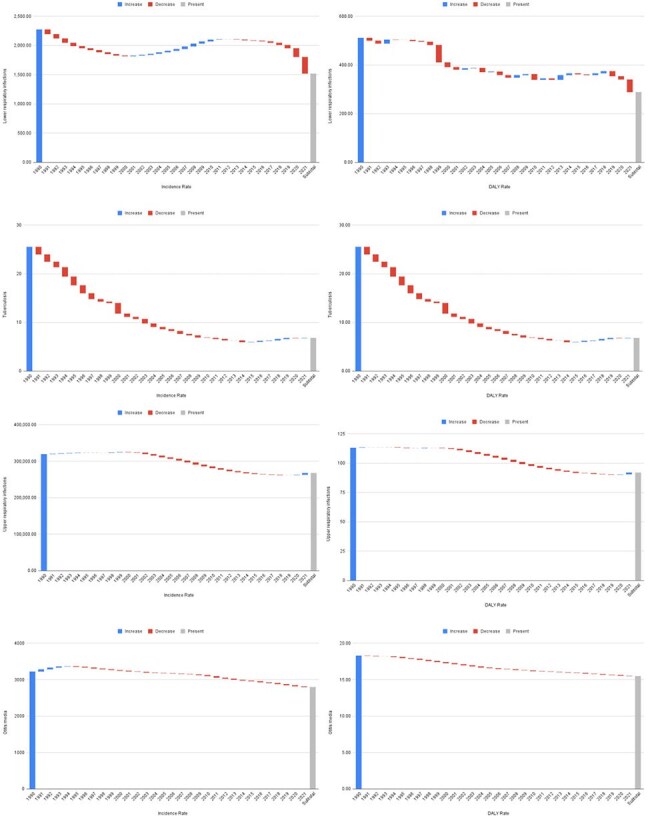
Waterfall charts showing temporal trends in incidence and DALY rates for major respiratory infections in the U.S. (1990–2021). Charts display increases (blue), decreases (red), and present values (gray) for lower respiratory infections, tuberculosis, otitis media, and upper respiratory infections. COVID-19 is excluded due to incomplete data beyond 2020. Rates are derived from the Global Burden of Disease (GBD) 2021 dataset.

**Methods:**

We examined 2021 data for five respiratory conditions—COVID-19, lower respiratory infections, otitis media, tuberculosis, and upper respiratory infections—across all U.S. states. Incidence data (age-standardized where available), DALYs, and risk factor attribution were collected and stratified by sex and geography. We assessed relationships between disease burden and key environmental and demographic risk factors, including child and maternal nutrition and air pollution.

**Figure 3:**
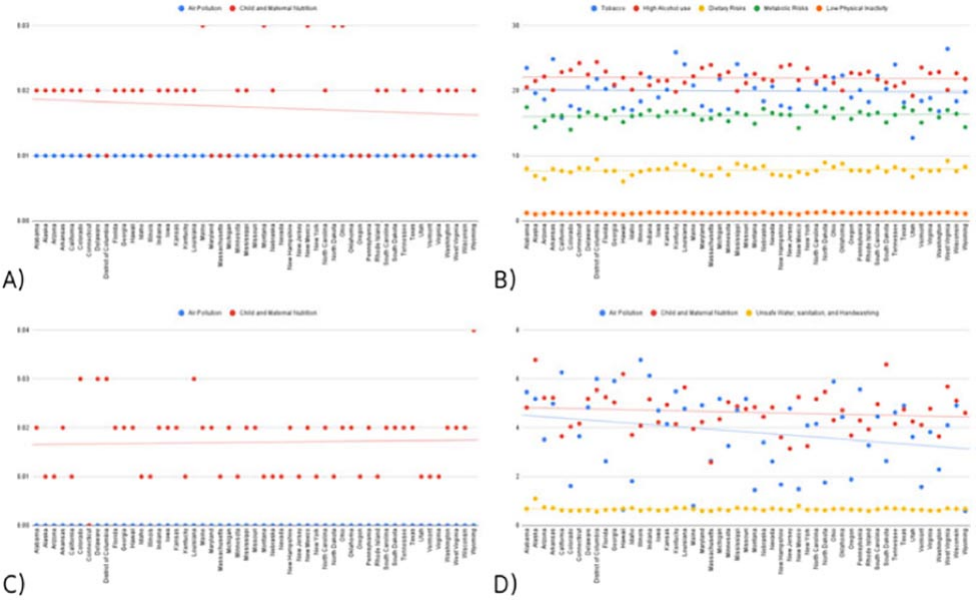
State-wise percentage of DALYs attributable to key risk factors for major respiratory infections in the U.S. (2021). Scatter plots depict the proportion of disease burden linked to specific risk categories for (A) upper respiratory infections, (B) tuberculosis, (C) otitis media, and (D) lower respiratory infections. Notable contributors include child and maternal nutrition, air pollution, and metabolic and behavioral risks. COVID-19 is excluded due to the absence of defined risk factor attribution in GBD 2021.

**Figure 4:**
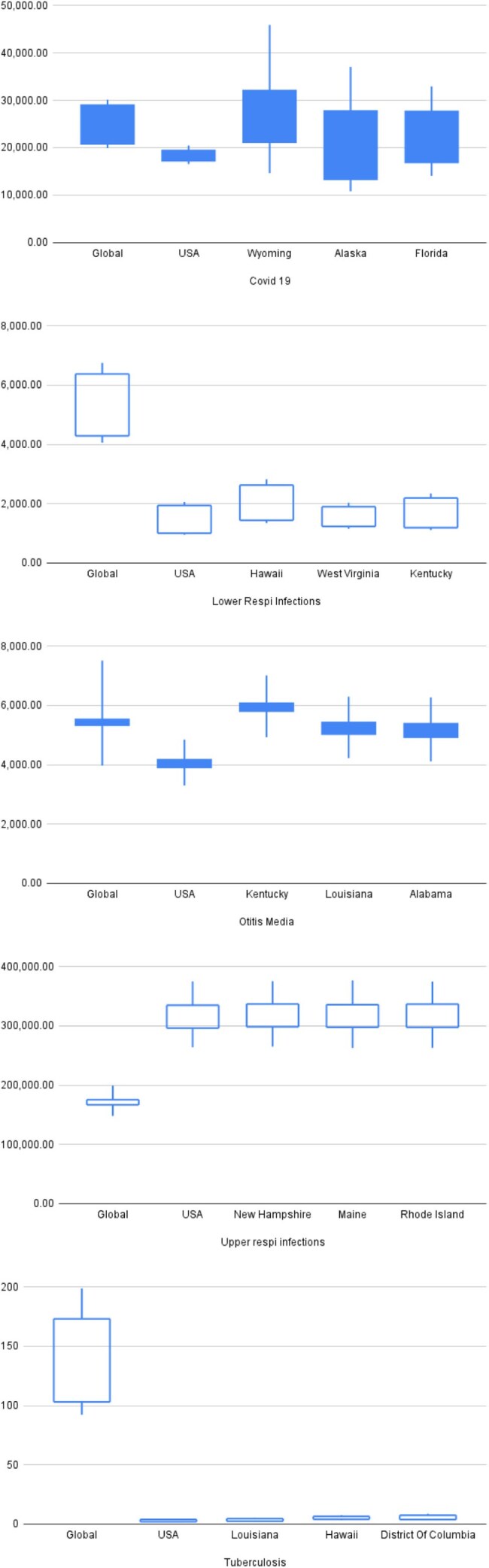
Incidence and uncertainty ranges for major respiratory infections across selected U.S. states and global estimates (2021). Boxplots display incidence rates with 95% uncertainty intervals for COVID-19, lower respiratory infections, otitis media, upper respiratory infections, and tuberculosis. Filled boxes represent an increase in incidence from 1990 to 2021; unfilled boxes indicate a decrease. The vertical range reflects the combined uncertainty across both time points by plotting the lowest lower bound and the highest upper bound from 1990 and 2021, ensuring the full range is represented. Global and national (U.S.) values are shown alongside the top three U.S. states with the highest burden for each condition. COVID-19 data represent only the period from 2020 to 2021 due to limited historical availability. Data source: Global Burden of Disease Study 2021.

**Results:**

Upper respiratory infections had the highest incidence nationally, while tuberculosis remained lowest. COVID-19 rates were highest in Wyoming, Alaska, and Florida; lowest in Hawaii, Vermont, and Washington. Lower respiratory infections were most prevalent in Hawaii, West Virginia, and Kentucky. Otitis media burden peaked in Kentucky, Louisiana, and Alabama. Child and maternal nutrition showed stronger correlations with disease burden than air pollution, particularly in states like Kentucky, Mississippi, Maine, and Oklahoma. Air pollution was weakly and uniformly associated with burden. Tuberculosis consistently had the lowest incidence across states.

**Conclusion:**

Gender and geography significantly shape respiratory infection burden in the U.S. These disparities call for gender-responsive, state-specific strategies grounded in local socio-environmental drivers.

**Disclosures:**

All Authors: No reported disclosures

